# Pilot Study on Feasibility of Sensory-Enhanced Rehabilitation in Canine Spinal Cord Injury

**DOI:** 10.3389/fvets.2022.921471

**Published:** 2022-06-14

**Authors:** Melissa J. Lewis, Jessica Bowditch, Brittany Laflen, Nicole Perry, Rachel Yoquelet, Stephanie A. Thomovsky

**Affiliations:** Department of Veterinary Clinical Sciences, Purdue University College of Veterinary Medicine, West Lafayette, IN, United States

**Keywords:** thoracolumbar intervertebral disc extrusion (TL-IVDE), disc herniation, neurorehabilitation, tactile-enhanced exercises, auditory-enhanced exercises

## Abstract

Physical rehabilitation is frequently recommended in dogs recovering from acute thoracolumbar intervertebral disc extrusion (TL-IVDE), but protocols vary widely. The objective of this study was to evaluate the feasibility of incorporating sensory-integrated neurorehabilitation strategies into a post-operative rehabilitation protocol in dogs with TL-IVDE. Non-ambulatory dogs with acute TL-IVDE managed surgically were prospectively recruited to this unblinded cross-over feasibility study. Eligible dogs were randomized to start with tactile-enhanced (artificial grass) or auditory-enhanced (floor piano) basic rehabilitation exercises performed twice daily for the first 4 weeks before switching to the opposite surface for the subsequent 4 weeks. Neurologic examination, open field gait scoring, girth measurements and an owner-completed feasibility questionnaire were performed at baseline and 2, 4, 6, and 8 weeks post-operatively. Twenty-four dogs were enrolled, 12 randomized to each order of exercises. Gait scores did not differ between the two groups at baseline, 4 or 8 week visits. All modified exercises could be performed and compliance was high. Adverse events potentially attributable to the study surface were mild, self-limiting and occurred in 2/24 dogs. The most common surface-related limitations were that the piano was slippery and that both surfaces were too short. The artificial grass was preferred by owners and dogs compared to the floor piano surface, but this was influenced by which surface was utilized first. Auditory and tactile modifications were feasible and safe to incorporate into a standardized rehabilitation protocol. This pilot study could prompt larger efficacy studies investigating the benefit of sensory-integrated rehabilitation in dogs with TL-IVDE.

## Introduction

Acute spinal cord injury (SCI) occurs frequently in dogs, commonly due to thoracolumbar intervertebral disc extrusion (TL-IVDE) (1, 2). A successful outcome after TL-IVDE in dogs is typically defined as resolution of pain and regaining independent ambulation and reasonable continence (3). Physical rehabilitation is frequently recommended to facilitate improvement (4, 5) with basic rehabilitation exercises consisting of cryotherapy, passive range of motion, massage, assisted standing and assisted walking (6–17). A variety of more intensive or multimodal protocols have been described in dogs with SCI, but the primary target of most rehabilitation therapy is the motor system (8, 11–23).

Sensory stimulation as a component of rehabilitation protocols in dogs is occasionally mentioned with descriptions of toe pinching, hair brushing, and utilizing different flooring surfaces (7, 10–12, 16, 19). However, details are limited regarding how they are incorporated and evidence to support such sensory-stimulating exercises is lacking in veterinary patients. In human stroke and SCI patients, sensory integration training has been shown to improve motor outcomes (24–29). This includes preferential stimulation of sensory fibers of peripheral nerves which promotes improved somatosensory processing and augments the effects of massed practice of motor skills (25, 26). Vibratory stimulation of specific muscle groups also provides enhanced proprioceptive feedback and improves motor function in people with incomplete tetraplegia (24) and stroke (28), and can be combined with other strategies such as using visual cues (30). Rhythmic auditory stimulation during gait training improves walking performance as does real-time auditory feedback of motor errors (27, 31–33). Visual strategies such as mirroring specific actions by an unaffected limb or providing real-time visual feedback during exercises appear to similarly promote improved motor learning and execution (27, 34). The benefit of sensory-based neurorehabilitation strategies in dogs with acute SCI is unknown.

The objective of this study was to evaluate the feasibility of incorporating tactile- and auditory-enhanced exercises into a standardized, post-operative basic rehabilitation protocol in dogs with TL-IVDE. We hypothesized that sensory-enhanced exercises would be simple to perform and well-tolerated by dogs recovering from acute TL-IVDE.

## Materials and Methods

### Study Animals

Client-owned dogs were prospectively recruited from the existing patient pool of the Purdue University Veterinary Hospital. Dogs had to have an acute SCI secondary to TL-IVDE resulting in non-ambulatory paraparesis or paraplegia with or without pain perception, be aged 1 to 10 years and weigh between 5 and 25 kg. The minimum weight limit was to ensure dogs were sufficiently large enough to produce sounds on the surface utilized for the auditory-enhanced exercises. The upper weight limit was chosen to facilitate adequate participation in exercises given length limitations of the study surfaces. Duration of neurologic signs had to be ≤7 days from onset of pain or pelvic limb deficits. Confirmation of TL-IVDE between the third thoracic and third lumbar vertebrae (T3-L3) was required based on computed tomography or magnetic resonance imaging. Decompressive hemilaminectomy was performed followed imaging confirmation of extruded disc material. The number of sites decompressed and whether durotomy or prophylactic fenestration were performed was at the discretion of the neurosurgeon.

Exclusion criteria included deafness, severe orthopedic or systemic disease, signs consistent with progressive myelomalacia on presentation, temperament (i.e., dogs not amenable to handling), or unwillingness to return for study rechecks. The study was approved by the Purdue Animal Care and Use Committee (protocol #201200210) and all owners provided informed consent at enrollment.

### Study Design

This study was a prospective, randomized, unblinded, cross-over design clinical trial. At 48 h post-operatively, eligible dogs were enrolled, stratified based on whether or not they had deep pain perception (deep pain positive or deep pain negative) and then assigned to one of two treatment groups in a 1:1 ratio using block randomization (groups of 4) with a cross-over design. Stratification ensured an even distribution of the most severely affected dogs (i.e., deep pain negative) between the treatment groups. Group 1 participated in tactile-enhanced neurorehabilitation for 4 weeks, then auditory-enhanced exercises for 4 weeks while group 2 participated in the opposite order of exercises. Dogs were evaluated at enrollment (baseline visit) and at 2-, 4-, 6-, and 8-weeks post-operatively.

### Study Procedures

**Figure 1:**
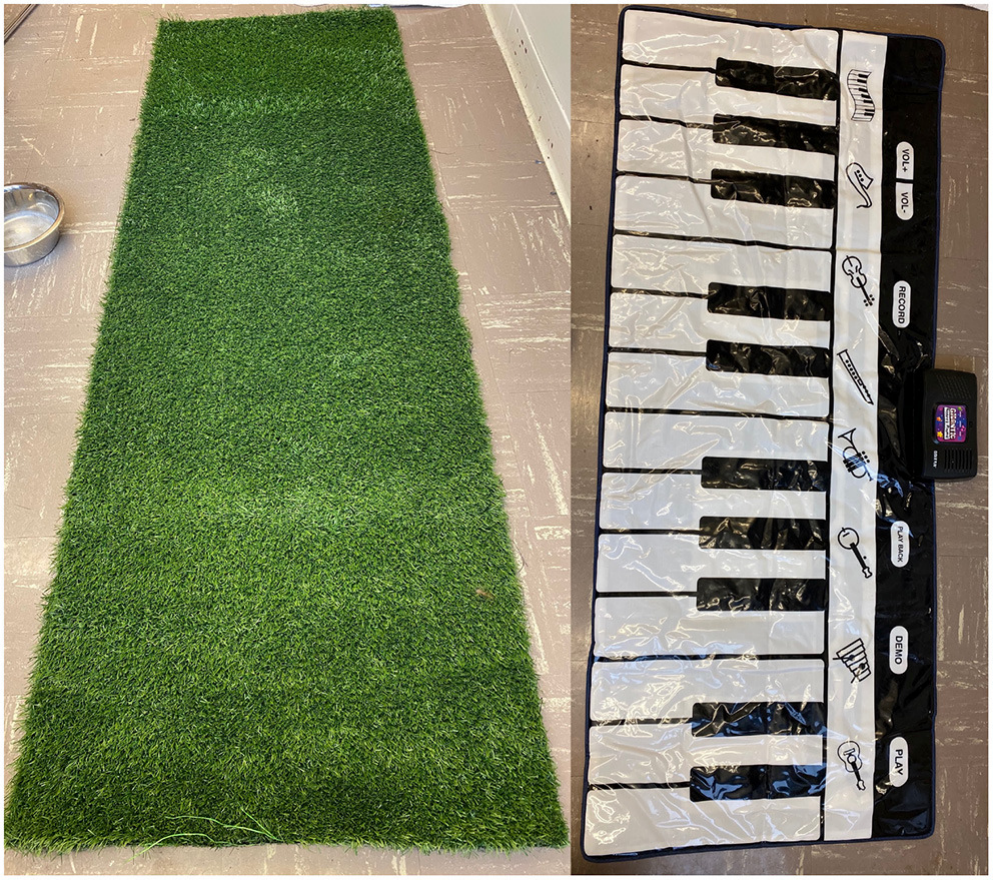
Examples of the artificial grass and floor piano used for sensory-modified exercises.

Touch (i.e., Artificial grass^1^) or sound (i.e., Floor piano mat^2^) modifications were incorporated into a standardized post-operative rehabilitation regimen which included: passive range of motion (PROM), assisted standing and weight-shifting and assisted walking. A 2-foot-wide by 6-foot-long strip of artificial grass and a 29-inch-wide and 70-inch-long child's floor piano were utilized as the tactile- and auditory-enhancements, respectively (Figure 1). The floor piano had a smooth surface. Passive range of motion was performed in a standing position where each pelvic limb was manually manipulated through range of motion (bicycling) at each joint to simulate limb movement during walking (Supplementary Video 1). The plantar surface of the paw was brought in contact with the study surface with each repetition, either touching the grass or touching and producing a sound on the floor piano. This was repeated 20 times per leg at each session. Assisted standing and weight shifting consisted of supporting the dog to stand squarely with all four limbs positioned on the study surface (Supplementary Video 2). The hips were then gently shifted from left to right and front to back for 5 min at each session. For PROM and assisted standing and weight shifting exercises, the use of the owner's hands, a sling, a physiopeanut or modified foam roller were utilized to provide hindquarter support as needed until dogs were able to support their own weight against gravity. Assisted walking was performed by repeatedly walking the dog on a leash across the study surface for 5 min per session (Supplementary Videos 3, 4). Hindquarter support was provided with the use of a sling or harness until dogs were independently ambulatory. When utilizing the floor piano for standing and walking exercises, the goal was to produce a sound each time the body weight was shifted or a step was taken, respectively.

All exercises were performed two times per day throughout the 8-week study period, including during initial hospitalization, starting 48-h post-operatively. Owners were sent home with the assigned study surface at the time of discharge from initial hospitalization and provided with the new study surface at the 4-week study visit. At the time of discharge and the 4-week recheck, owners were instructed verbally and via demonstrations on how to perform each exercise including how to use and incorporate each study surface.

At each study visit, the following procedures were performed: physical and neurologic examinations, open field gait scoring, body and limb circumference measurements, and an owner-completed questionnaire. Neurologic examination consisted of evaluation of mentation, gait, cranial nerves, postural reactions, spinal reflexes, presence of spinal hypersthesia, pain perception and continence. Gait was classified as normal, ambulatory paraparesis, non-ambulatory paraparesis or paraplegia. Ambulation was defined as being able to take at least 10 consecutive weight-bearing steps without falling. Gait was also scored using the validated 0-12-point open field gait scale (OFS) (35, 36). A Gulick-type 2 tape measurement device^3^ was utilized for all circumference measurements and performed by trained personnel. Three circumference measurements were performed in triplicate as previously reported including caudal trunk girth and right and left thigh girth (37). Briefly, the caudal trunk measurement was performed in a standing position with girth measured around the abdomen just cranial to the inguinal folds. Limb girth measurements were performed in lateral recumbency with the circumference of the upper limb measured at 50% of the length of the femur from the greater trochanter. A questionnaire was completed by owners at each study visit (Supplementary Figure 1). The questions focused on compliance and feasibility regarding the ease of completion, patient tolerance and any adverse effects associated with the rehabilitation exercises or the study surface.

### Statistical Analysis

As a pilot, feasibility study, a power analysis was not performed. A minimum sample size of 20 dogs was planned with the aim of providing sufficient preliminary data on our methods. Descriptive statistics were utilized to summarize enrollment and feasibility data acquired in this study. Triplicate girth measurements were averaged to provide a mean value for each dog at each visit. To account for dogs of varying size and conformation, these measurements were expressed as a percentage of the baseline values. Mean OFS scores and girth measurements at baseline, 4 and 8 weeks post-operatively were compared using a *t*-test to look for any differences between groups. *P* < 0.05 was considered significant.

## Results

Twenty-four dogs were enrolled with a mean age of 4.4 years (SD 2.2) and mean body weight of 9.4 kg (SD 4.5) at baseline. Breeds included 12 dachshunds, four mixed breed dogs, three French bulldogs, and five breeds represented by two or fewer dogs. Mean duration of neurologic signs prior to enrollment was 3.2 days (SD 1.2), accounting for a 48-h interval from presentation and surgery to enrollment. Twelve dogs including twoparaplegic with absent pain perception were randomized to start with exercises incorporating the artificial grass (Group 1). This group had a mean age of 3.8 years (SD 2), mean body weight 11 kg (SD 4.9) of and mean duration of signs of 3.2 days (SD 1.1). Twelve dogs including 3 paraplegic with absent pain perception were randomized to start with the floor piano (Group 2). This group had a mean age of 5 years (SD 2.4), a mean body weight of 7.7 kg (SD 3.6) and a mean duration of signs of 3.2 days (SD 1.2). Group 1 was significantly heavier than group 2 (*p* = 0.04), but no significant differences were identified between groups with regard to age or duration of signs (*p* > 0.05).

All dogs were diagnosed with TL-IVDE between T10 and L3 and underwent decompressive surgery. Surgical plan including number of sites decompressed and prophylactic fenestration varied between cases. No intra-operative complications were encountered. One dog required a second decompressive surgery 3 days after the first due early re-herniation of disc material resulting in paraplegia with intact pain perception. This dog was enrolled in the study 48 h after the second surgery (with a neurologic status of paraplegia with intact pain perception) and no physical rehabilitation exercises were performed (of any kind) until enrollment and randomization. Nineteen dogs completed all study visits (nine in group 1, 10 in group 2), one dog completed three of the four rechecks (missed 6-week visit), two dogs completed two of four rechecks (missing 6- and 8-week rechecks), one dog completed only the 2-week recheck. One additional dog was euthanized within 1 week after the baseline visit (due to lack of neurologic improvement).

At baseline, seven dogs were non-ambulatory paraparetic (two in group 1, five in group 2), 12 were paraplegic with intact pain perception (eight in group 1, four in group 2) and five were paraplegic with absent pain perception in their pelvic limb toes and tail base (two in group 1, 3 in group 2). Of the dogs with available follow-up data, 16 dogs were ambulatory by 2 weeks, 17 dogs were ambulatory by 4 weeks and 18 dogs were ambulatory by 8 weeks or sooner. At study completion, 4 dogs remained non-ambulatory, of which 3 had persistently absent pain perception with varying degrees of pelvic limb motor. Gait scores across study visits are outlined in Table 1. There were no significant differences identified in OFS scores between groups at baseline, 4 and 8-week study rechecks (*p* > 0.05).

**Table 1:** OFS scores at each study visit.

	**OFS Scores Median (range)**		
**Study visit**	**All dogs**	**Group 1 (grass, then piano)**	**Group 2 (piano, then grass)**
Baseline (*n* = 24)	0 (0–4)	0 (0–1)	0 (0–4)
2-week (*n* = 23)	6 (0–9)	5.5 (0–9)	7 (1–9)
4-week (*n* = 22)	8 (2–11)	7 (2–11)	8 (2–11)
6-week (*n* = 19)	8 (2–12)	8 (3–12)	8 (2–11)
8-week (*n* = 20)	9 (3–12)	9 (3–12)	9 (3–11)

*OFS, open field scale*.

Proprioception (paw placing) was absent in all dogs at baseline. By study completion, proprioceptive placing had completely normalized in five dogs, including two dog from group 1 and three dogs in group 2, but remained delayed or absent in the remainder. Mean girth measurements expressed as a percentage of baseline values are outlined in Table 2. Caudal trunk girth was decreased at 4 weeks compared to baseline in both groups; by 8 weeks post-operatively, this had returned to baseline in group 2 but remained lower in group 1. Left and right thigh circumference measurements did not demonstrate clear trends. Changes were generally small and no significant differences between groups over time were identified (*p* > 0.05).

**Table 2:** Mean thigh and body girth measurements at the 4- and 8-week study visits, expressed as a percentage of baseline values.

	**Group 1 (grass, then piano)**			**Group 2 (piano, then grass)**		
**Study visit**	**Caudal trunk girth % (SD)**	**Left thigh girth % (SD)**	**Right thigh girth % (SD)**	**Caudal trunk girth % (SD)**	**Left thigh girth % (SD)**	**Right thigh girth % (SD)**
Baseline	100	100	100	100	100	100
4-week	94.7 (8.8)	102.7 (8.2)	98.8 (14.0)	94.2 (8.5)	104.2 (12.4)	100.3 (11.7)
8-week	95.6 (4.1)	98 (14.4)	100.4 (13.3)	100.9 (8.0)	103 (13.1)	103.0 (12.9)

Adverse events during the course of the study occurred in 5/24 (21%) dogs, including two dogs in which it was considered attributable to the tactile or auditory modifications. In one dog, the piano noise was noted to be particularly aversive and another dog developed a superficial abrasion on the dorsum of the right pelvic limb paw during the study period when using the artificial grass. Neither event required intervention and the exercises were continued by both owners. Additional adverse events reported by owners in three dogs were related to their neurologic status. This included one dog initially paraplegic deep pain negative that regained pain perception by discharge but was euthanized at 1 week post-operatively due to lack of recovery of function. Another dog initially recovered uneventfully but had a recurrence of paraplegia at 4.5 weeks post-operatively due to a presumptive re-herniation. This dog was managed conservatively and had regained independent ambulation by the next study visit. A third dog was noted by the owner to be intermittently, mildly painful when performing the daily exercises during the first 2 weeks post-operatively. No adjustments to the dog's analgesic protocol were required.

In 20/24 (83%) dogs, all modified exercises were performed as instructed, while owners of four dogs reported that they were unable to perform all of the exercises twice daily at some point during the study period. In 3 dogs, this was reported for a single 2-week period while one owner reported incompletely performing the exercises over a duration of 4 weeks. The reasons cited included being busy or other scheduling conflict in 3 dogs and fear of worsening status in one dog that suffered a presumptive re-herniation.

All exercises were able to be performed with the modifications. However, ease of use varied between dogs and surfaces. Summarizing owner-reported feasibility across all study visits in which data was available, 13/22 (59%) owners reported that the grass was easy to use while 9/22 (41%) owners reported at least once that use was associated with mild difficulty. Six of 20 (30%) dog owners reported that the piano was easy to use while 14/20 (70%) owners rated on at least one occasion that this surface was mildly difficult (12/20, 60%) or hard to use (2/20, 10%). No trends over time during the 8-week study period regarding ease of use for each surface were identified.

Feedback relayed via the questionnaire could be subdivided into comments that were related vs. unrelated to the surfaces. Positive experiences related to the the artificial grass were reported in two dogs including one dog that ‘loved the grass' and another where it ‘reminded the dog of being outside.' Positive experiences related to the floor piano were also reported in two dogs including one dog that ‘liked making noises' and another where it ‘seemed like a game.' The most common surface-related limitations or negative experiences reported were that the piano was too slippery and both the grass and piano were too short, especially when performing the walking exercises. These were noted in ~25 and 15% of questionnaire responses, respectively. Less than 10% of responses indicated that dogs initially disliked or were scared of the artificial grass texture or the piano noises, though all were reported to get used to it on subsequent responses. One dog weighing 6.4 kg was noted to be too small to consistently make noise on the floor piano when performing the exercises, though this was not specifically reported in the seven other dogs weighing the same or less (5.4–6.4 kg) than this dog.

There were non-surface related comments provided in about 27% of questionnaire responses and these were generally attributable to behavioral limitations associated with performing the exercises. The most commonly reported behavioral difficulties included that the dog got distracted or tried to move away from the owner and the designated surface/exercise area, that the dog was not cooperative when performing the exercises, that the dog became bored or frustrated during the 5-min sessions for each exercise, and that the duration of the exercises was too long. Owners also noted that these limitations became more frequent or problematic to overcome as their dogs improved and regained more pelvic limb function.

Of the 20 dogs that completed an 8-week study visit, owners of 14 dogs preferred the grass, three preferred the piano, two indicated an equal preference for both and one did not provide an answer. Eleven owners indicated that their dog preferred the grass, two thought their dog preferred the piano, five indicated their dog liked both surfaces equally, 1 owner reported that their dog disliked both surfaces equally, and one owner did not provide an answer. The surface preferences varied by group (Table 3). When starting with the piano first (group 2), 9/10 owners and 9/10 dogs preferred the grass. However, when starting with the grass first (group 1), 5/9 owners and 2/9 dogs preferred the grass.

**Table 3:** Owner and dog surface preference stratified by group allocation.

**Dog number**	**Group 1 or 2**	**Owner surface preference**	**Dog surface preference**
1	Group 1	Piano	Piano
2	Group 1	Piano	Piano
3	Group 1	Grass	Both
4	Group 1	Grass	Both
5	Group 1	Both	Both
6	Group 1	Both	Both
7	Group 1	NA	NA
8	Group 1	NA	NA
9	Group 1	Grass	Grass
10	Group 1	NA	NA
11	Group 1	Grass	Grass
12	Group 1	Grass	Both
13	Group 2	Grass	Grass
14	Group 2	Grass	Grass
15	Group 2	Grass	Neither
16	Group 2	Grass	Grass
17	Group 2	NA	NA
18	Group 2	Grass	Grass
19	Group 2	Grass	Grass
20	Group 2	Grass	Grass
21	Group 2	Grass	Grass
22	Group 2	Grass	Grass
23	Group 2	Piano	Grass
24	Group 2	NA	NA

*Group 1, grass first; Group 2, piano first; NA, data not available*.

## Discussion

This is the first study specifically investigating sensory-enhanced rehabilitation exercises in dogs recovering from acute TL-IVDE. Our results demonstrated that simple auditory and tactile modifications were feasible and safe to incorporate into a standardized rehabilitation protocol. While both surfaces were generally well-tolerated, dog behaviors independent of the surface contributed to challenges in performing the exercises during the study period. This preliminary information could be used to design larger efficacy studies investigating the benefit of sensory-enhanced neurorehabilitation and to continue to optimize rehabilitation protocols in this population.

Incorporating two different, readily accessible surfaces, a piece of artificial grass or a child's floor piano, we provided a simple means to enhance sensory feedback as part of a basic post-operative rehabilitation protocol consisting of PROM, assisted weight shifting and assisted walking. There is very limited detail from prior studies in veterinary patients regarding how exercises with a sensory component are incorporated (8, 10–12, 16, 19). Importantly, sensory stimulation exercises in the post operative veterinary neurologic patient typically center on the owner or rehabilitation professional stimulating the patient's feet, with activities like toe pinching, tickling or rubbing having been described (6, 8, 10, 11). This study was different in that the sensory stimulation was initiated by the patient's foot landing on the artificial grass or floor piano surface, and therefore, incorporated into the exercises themselves. Specific sensory-integrated techniques are utilized in people with SCI as well as other conditions such as stroke (24–28). These approaches allow intact sensory systems (e.g., auditory system) to provide appropriate input in the form of specific sensory cues during various motor training tasks to aid in recovery or compensation of an impaired sense (e.g. propioeption) after injury (38, 39). Music, through its ability to stimulate memories and the so-called memory-movement connection, has also been described as a rehabilitation strategy to promote muscle memory and enhance movement (40). A variation on this, known as cognitive multisensory rehabilitation, uses multisensory input to restore brain connectivity relating to awareness and pain perception that is impaired after SCI (41). Reported benefits include rebuilding the mind-body connection, improving body awareness and reducing neuropathic pain (41). Sensory-integrated neurorehabilitation approaches have also been associated with improved motor outcomes and enhanced overall functional recovery (24–28, 34, 38, 39).

Given the potential benefits demonstrated by human neurorehabilitation studies combined with the dearth of information in veterinary SCI patients, our rationale was to explore novel sensory stimulation integrated into a standardized rehabilitation protocol. We demonstrated that the tactile or auditory adaptations were simple to apply and that the modified exercises could be performed by veterinary professionals and owners with no specific training or skills. Additionally, the dogs of this study with severe SCI secondary to TL-IVDE were amenable to both the tactile and auditory sensory stimulation, regardless of their neurologic status or recovery trajectory. Patients who were paralyzed or severely paretic and also those who regained independent walking tolerated both types of sensory stimulation. In addition to feasibility, no substantial adverse events directly attributable to the surfaces or exercise modifications were encountered. Initiation of exercises at 48 h post-operatively was well-tolerated which is consistent with other clinical trials of post-operative physical rehabilitation (8, 17).

While this study was not designed to evaluate efficacy, we utilized open field gait scoring, proprioceptive placing and caudal trunk and thigh girth measurements to evaluate outcomes. No significant differences were identified in these measures between the two treatment groups at 4 or 8 weeks post-operatively, but there was no control group. In addition to incorporating controls and blinding evaluators, outcome measures that can quantify the potential influence of a sensory-integrated approach would help to evaluate the efficacy of our methods. For example, quantitative sensory thresholds have been established in dogs with acute SCI (42–45) and could be used to evaluate if sensory stimulation aids in re-establishing more normal thresholds after injury. Additionally, the F-response and H-reflex provide information on motor neuron pool excitability in dogs with acute and chronic SCI (46, 47). These electrodiagnostic tests could help to objectively determine if sensory enhanced exercises provide appropriate afferent input to positively impact motor neuron pool excitability and, in turn, contribute to motor recovery. Body weight distribution has also been quantified in dogs after SCI (37) and could be used with girth measurements and proprioception to determine if sensory interventions intended to improve limb awareness affect pelvic limb weight distribution and muscle mass. Evaluation of nuanced gait parameters such as velocity, cadence and stride length are utilized in human stroke patients undergoing rehabilitation (48, 49). Treadmill-based stepping and coordination scores are validated in dogs with SCI and could be similarly utilized to objectively measure rehabilitation progress (50). Tailored outcome measures could provide important evidence of a link between an enhanced, integrated sensory environment and improved functional recovery after SCI in dogs.

While all modified exercises were feasible, owner feedback highlighted several limitations regarding ease of completion. The short length of both the artificial grass and floor piano as well as the slippery surface of the piano were recurrent comments. This suggests that additional refinement of our techniques for incorporating sensory modifications is needed to improve feasibility, ensure appropriate compliance and optimize the potential benefit. Future adjustments to the tactile-enhanced exercises could include using a textured surface of longer length or incorporating expanded tactile modifications, including taking advantage of natural outdoor surface variations on assisted walks. Providing varied terrain (e.g., tall grass, gravel, etc.) has been mentioned for dogs with recovering from disc herniation (12), but specific protocols have not been established. Providing auditory feedback via an alternative method other than the floor piano might eliminate the body size restrictions and the need to utilize a slippery surface in non-ambulatory dogs. Sound, delivered in the form of musical notes with beat and rhythm, might also be most advantageous in the later stages of gait refinement and coordination as compared to earlier stages of regaining movement after SCI (32, 33, 51). Rhythmic auditory stimulation has been incorporated into rehabilitation programs following a variety of conditions in people including SCI, stroke and movement disorders (48, 51–54). Rhythmic auditory stimulation is based on the idea of entrainment, in which rhythmic patterns produced by sounds or music directly improve movement timing and efficiency (55). This sensory technique can be combined with treadmill training to improve gait speed and balance and could be adapted for dogs. Therefore, it is possible that timing of certain types of sensory stimulation is important and that auditory integration might be more useful once a dog is more functional or even ambulatory. Another alternative strategy could be utilizing auditory cues to highlight mistakes. This has been used to improve motor performance in people with stroke (31). Future larger scale efficacy studies are warranted comparing different types of tactile and auditory stimulation to each other in an ongoing effort to optimize rehabilitation protocols, including how best to incorporate sensory integration in both the hospital and home-care settings.

Owner reported compliance was generally high throughout the study, but owner feedback also commonly focused on non-surface related issues. This included things such as dog boredom, distractability or lack of cooperation for performing the exercises, which were reported to worsen as pelvic limb function improved. In prior studies in which at-home rehabilitation regimens are recommended (10–12, 17), sparse information is provided regarding if there was adherence to protocols or if any challenges were encountered in the proper execution of the exercises by owners. We do not have baseline data on compliance in performing these exercises without the sensory modifications. However, our results underscore that dog behavior could substantially impact proper performance of prescribed exercises and owner perception of the recovery process and willingness to engage in at-home protocols. In people with SCI, explicitly outlining daily tasks and recommended exercises provides clear expectations, structure and consistency which in turn improves compliance with rehabilitation participation (56). While there are obvious differences between motivating a dog vs. onself to participate in at-home rehabilitation, our findings support that behavioral factors should be considered when developing an at-home therapy regimen. Additionally, dynamically acquiring and responding to owner feedback might improve both owner and dog participation.

At study completion, 70% of owners preferred the grass and 55% of owners thought their dogs also preferred the grass while just 15% of owners and 10% of dogs preferred the piano. While this might support a true preference for the artificial grass surface, there was a discrepancy based on whether dogs were randomized to group 1 (grass first) or group 2 (piano first). When rehabilitation exercises were initially performed on the piano, the vast majority of the dogs and owners preferred grass as compared to the piano. The opposite, however, was not the case; when dogs used grass for the first half of the study, owner preference for grass over the piano was less decisive and dog preference was essentially equally spread between the surfaces. Therefore, the role of the order of surfaces and the dogs' neurologic status during the recovery period must be considered as factors impacting this preference. Similar to previously published data on recovery rates (57), the majority of dogs in this study regained independent ambulation within the first 4 weeks post-operatively. Thus, when pelvic limb motor function was worse during the first half of the study, the smooth surface of the floor piano likely made it more difficult to perform the exercises while the textured surface of the artificial grass could have provided better traction. This might help to explain why owners of dogs randomized to group 2 (piano first) more strongly favored the artificial grass. Once greater functional status was achieved, the slippery nature of the piano might have been less of a detractor and could account for the more even distribution of preference in dogs randomized to group 1 where the piano was not utilized until the latter half of the study. Another potential contributing factor is that group 2 was significantly lighter than group 1. Being lighter makes it harder to make noise on the floor piano and could exacerbate the lack of traction further influencing the preference toward the artificial grass.

Similarly, all owners whose dogs completed the study but did not regain ambulation during the 8 weeks of follow-up, preferred the grass to piano. In these more severely affected dogs, traction and support from the artificial grass might have facilitated more easily performing exercises compared to the smooth surface of the piano. While pelvic limb tone and ability to bear weight against gravity improved over the course of the study among the dogs that remained non-ambulatory, this might not have been enough to improve the ease of completion of exercises on the floor piano (relative to the grass) and influenced the owner's preferred surface.

Overall, this pilot project demonstrated that sensory integrated rehabilitation was feasible in dogs recovering from severe SCI and provides a framework to continue to investigate multisensory rehabilitation protocols incorporating visual, auditory, tactile, or somatosensory stimulation, or a combination of approaches. These preliminary results will be useful to design future, larger scale efficacy studies on sensorimotor integration into intensive, staged rehabilitation protocols in dogs recovering from SCI.

## Data Availability Statement

The raw data supporting the conclusions of this article will be made available by the authors, without undue reservation.

## Ethics Statement

The animal study was reviewed and approved by Purdue University Institutional Animal Care and Use Committee (protocol # 201200210). Written informed consent was obtained from the owners for the participation of their animals in this study.

## Author Contributions

MJL and SAT participated in study design, data acquisition and analysis, manuscript preparation, and editing and review. JB, BL, NP, and RY participated in data acquisition and analysis and manuscript editing and review. All authors contributed to the article and approved the submitted version.

## Funding

This project was funded by a grant from the American Association of Rehabilitation Veterinarians (AARV) and the Purina Institute.

## Conflict of Interest

The authors declare that the research was conducted in the absence of any commercial or financial relationships that could be construed as a potential conflict of interest.

## Publisher's Note

All claims expressed in this article are solely those of the authors and do not necessarily represent those of their affiliated organizations, or those of the publisher, the editors and the reviewers. Any product that may be evaluated in this article, or claim that may be made by its manufacturer, is not guaranteed or endorsed by the publisher.

## Footnotes

^1^Greenline Jade 50 15ft Wide x Cut to Length Artificial Grass, Home Depot, homedepot.com.

^2^Click N' Play Gigantic Keyboard Play Mat 70 x 29 inches, Amazon, amazon.com.

^3^Gulick II Tape Measure, Fitness Mart, GaysMills, WI.
