# Supplementary figures and images for: Pilot Study on Feasibility of Sensory-Enhanced Rehabilitation in Canine Spinal Cord Injury
Source: Front Vet Sci. 2022 Jun 14;9:921471. doi: 10.3389/fvets.2022.921471 (PMC9237616; doi:10.3389/fvets.2022.921471)

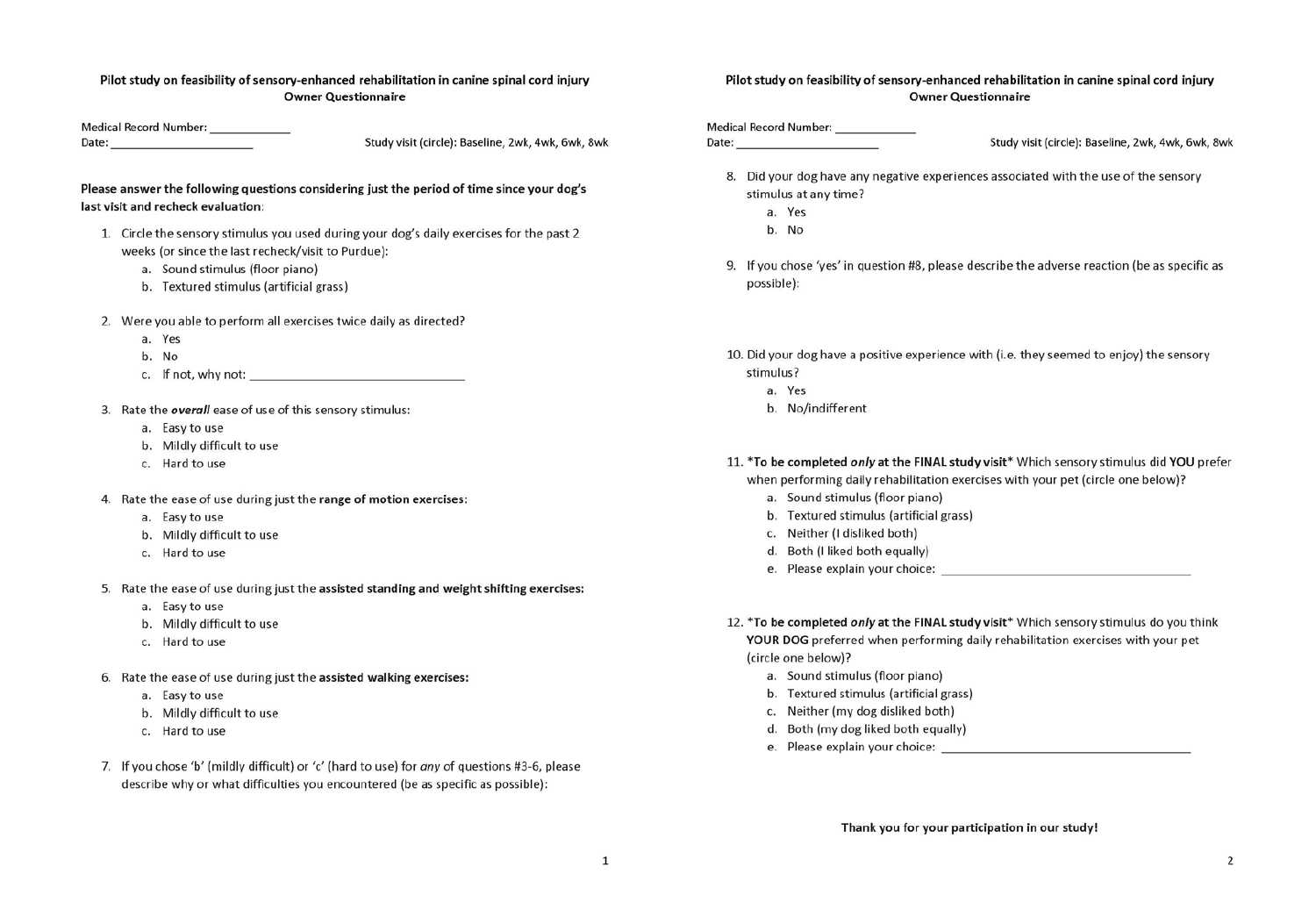

Supplement: Supplementary file 5 [file Image_1.TIF]
